# Analysis of Volatile Flavor Compounds of Corn Under Different Treatments by GC-MS and GC-IMS

**DOI:** 10.3389/fchem.2022.725208

**Published:** 2022-07-04

**Authors:** Kangyi Zhang, Lingling Gao, Can Zhang, Tao Feng, Haining Zhuang

**Affiliations:** ^1^ Institute of Agricultural Products Processing, Henan Academy of Agricultural Sciences, Zhengzhou, China; ^2^ School of Perfume and Aroma Technology, Shanghai Institute of Technology, Shanghai, China; ^3^ School of Health and Society Care, Shanghai Urban Construction Vocational College, Shanghai, China

**Keywords:** corn, gas chromatography ion mobility spectrometry, gas chromatography-mass spectrometry, flavor, treatments

## Abstract

To establish a rapid and accurate method for detecting volatile components of corn, which will guide the production of corn products beloved by consumers. The fingerprints of corns under different treatments, including native, washing, blanching, precooling, freezing, steaming, boiling, frying, and freeze-drying, were depicted via gas chromatography ion mobility spectrometry (GC-IMS) and gas chromatography-mass spectrometry (GC-MS). It was found via the Venn diagram and relative odor activity value (ROAV) that n-hexanal, 1-octene-3-ol, decylaldehyde, and 2-pentylthiazole could be the key flavor compounds present in corns. In addition, according to volatile fingerprint characteristics and the aroma profile of sensory evaluation, it was found that corns could be divided into four categories, which was consistent with the results of GC-IMS. Also, the results of the sensory panel showed that steamed, boiled, and fried corns were much more popular than corns under other treatments with the panel. The results indicated that a rapid method to classify products was established by GC-IMS. A suitable processing technology could produce a specific flavor, and further refined research might be focused on finding the best way to process corns.

## Introduction

Corn (*Zea mays L.*), known as “golden food”, is an annual crop that belongs to the grass family, and it is an important food crop around the world. It is native to Central and South America and widely distributed in the United States, China, Brazil, and other countries (Plate and Gallaher, 2005). Corn (especially fresh corn) not only contains starch (23.08%), protein (12.96%), fat (4.31%), fiber (14.95%), and other nutrients but is also rich in calcium, iron, magnesium, selenium, vitamins, carotene, and nicotinic acid (Oboh et al., 2010). Corn has certain medicinal effects, such as prevention and treatment of cardiovascular diseases, promotion of intestinal wall peristalsis, prevention of constipation, anti-oxidation, anti-cancer, etc. (Abdulrahaman and Kolawole, 2006). With the enhancement of health awareness, corn is becoming more and more popular with people and its consumption is increasing rapidly. Studies have found that the appearance and flavor of corn are the key factors affecting consumer acceptance and volatile flavor compounds play an important role in the acceptability of corn (Li et al., 2016). As for the detection of flavor compounds in corn, Legendre et al. (1978) had used GC-MS to detect flavor compounds in corn as early as 1978. In addition, the volatile flavor compounds of some frozen and fresh corn products were analyzed, and the key aroma compounds which were identified include 2-acetyl-1-pyrroline and 2-acetyl-2-thiazoline (Buttery, et al., 1994). Our team has studied the effects of three drying methods (oven drying after boiling, baking, and freeze-drying) on aroma components of fresh corn, and comprehensive analysis showed that the fresh corn dried by freeze-drying and oven drying after boiling had better retention of aromatic compounds. There are many treatments to process corn including washing, blanching, precooling, and freezing in the fresh-keeping stage as well as steaming, boiling, frying, and freeze-drying in the deep processing stage. However, there are few studies related to flavor analysis of corns under various treatments.

In recent years, GC-IMS and GC-MS are extensively applied to investigate the volatile compounds of cereal crops, such as rice (Błaszczak et al., 2007; Xia et al., 2017), wheat (Pico et al., 2016), barley (Cramer et al., 2005), oat (Sides et al., 2001), millet (Liu et al., 2012), and buckwheat (Janeš, et al., 2012). GC-IMS was used to detect the volatile compounds of white and yellowed rice, and a yellowing process monitoring method during rice storage was developed based on GC-IMS (Zhang et al., 2020). Mohamed Farag et al. (2019) delineated the sensory and nutritive quality of grilled corn using a metabolomics approach as revealed via SPME and GC-MS analyses and improved the taste and flavor of grilled corn reasonably based on detailed chemical analyses. GC-MS was used to identify 78 volatile compounds from cooked brown rice and germinated brown rice (Xia et al., 2017). Błaszczak et al. (2007) investigated the impact of high hydrostatic-pressure processing on the volatile profile of cooked Japonica rice and Jasmine rice by GC-MS, which could be a suitable alternative to traditional pretreatment for improving the flavor in cooked rice.

GC-MS is a widespread and effective method based on solid-phase microextraction, gas-phase separation, and mass spectrometry for analyzing volatile compounds in food samples. GC-MS qualitative analysis relies on the calculation of retention index; volatile compounds were identified by comparing their retention times, linear retention indices, and mass spectra with those of the authentic standards analyzed under identical chromatographic conditions (Rodríguez-Maecker et al., 2017; Ghader et al., 2018). The basic principle of GC-IMS technology is that the volatile components in the sample were pre-separated by a chromatographic column and eluted directly to the IMS ionization chamber for ion migration analysis (Nölting 2010; Wang et al., 2017). The hyphenated approach skillfully combines the high separation of GC with the high sensitivity of IMS and achieves the analytical effect of mutual superposition of advantages. The three-dimensional spectrum results of HS-GC-IMS contain the retention time, drift time, and signal intensity, which improve the accuracy of the qualitative analysis. GC-IMS not only has a stronger separation ability and lower cost of the instrument but also can avoid the disadvantages of complicated operation and difficulty in analysis. By combining the gas-phase analysis technology and ion mobility spectroscopy ingeniously and organically, GC-IMS can analyze the test results quickly, simply, intuitively, and accurately and does not need sample pretreatment and heating process, which can retain the original flavor of the sample to the greatest extent and make the analysis results more authentic and convincing (Kafle et al., 2016; Puton and Namieśnik, 2016; Zhang et al., 2016; Gallegos et al., 2017). Though there are a number of GC-MS applications carried out with no sample pretreatment and low-temperature heating processes, it is not suitable for our corn samples, which require a 50 min heat treatment for GC-MS in this study. However, some European or international decisions and regulations (e.g., SANTE-2020-12830; Regulation EU/2021_808 repealing 657_2002) recommended that methods based on chromatographic analyses without the use of mass spectrometric detection are not suitable on their own for use as confirmatory methods. Moreover, due to its limitations in the reliable quantitative analysis, GC-IMS cannot yet replace GC-MS, which remains the gold standard for food flavor analysis. The combinations of GC-IMS with other techniques have been reported to be highly effective for the analysis and characterization of volatile compounds (Fan et al., 2020). For example, Guo et al. (2018) investigated the effect of hot air treatment on volatile compounds of Tricholoma matsutake by GC-IMS and GC-MS. The combination of different techniques discloses more comprehensive, reliable, and scientific information on food aroma. However, to the best of our knowledge, monitoring the variations of volatile flavor during cereals processing via the combination of GC-MS and GC-IMS is rarely reported, thus, GC-MS combined with GC-IMS as an additional separation method was used in this design for achieving a better separation of isomers.

The study is primarily focused on the following three objectives: 1) To establish a rapid and accurate method for identifying volatile components of corn, which will guide the production of corn products fond to consumers; 2) To provide a novel methodology to investigate the relationship between flavor compounds and various processed technology for various cereals; 3) To establish a visualized method for identifying volatile compounds in corn as well as a creative compensation way for classifying distinct corn products.

## Materials and Methods

### Materials

The corn used in the experiment was sweet corn, and it was provided by the Henan Academy of Agricultural Sciences (Zhengzhou, Henan, China). The picking time and maturity of corn are similar (picking time is 2019) and the preservation method is cold storage. The different processing methods are as follows: native (fresh corn untreated), washing (rinsed the natural corn with water at 25°C three times gently), blanching (washed-corn boiled in boiling water for 2 minutes), pre-cooling (refrigerate after blanching: 4°C, 2 h), freezing (freeze after blanching: -18°C, 2 h), steaming (washed corn steamed for 15 min), boiling (washed corn boiled in boiling water for 15 min), frying (washed corn fried for 20 min until the corn produces flavor), and freeze-drying (washed corn dried by vacuum freezing). Fresh, plump, and uniform corn kernels were selected and divided into nine equal portions randomly, and then they were processed according to the aforementioned methods. Treated samples were ground immediately and weighed accurately for flavor identification, and three replicates were performed for each treated sample.

### GC-IMS Determination Conditions

Gas chromatography combined with ion mobility spectrometer (GC-IMS) (G.A.S, Germany) was used for the rapid measurement of volatile compounds. The samples were measured using a static headspace sampling method with an automatic sampler unit (CTC, Switzerland) followed by the GC-IMS analysis (FlavourSpec, G.A.S, Germany) (Denawaka et al., 2014). According to Wang et al. (2020), the GC system was equipped with an FS-SE-54-CB capillary column (15 mm × 0.53 mm × 1 μm), and a heated splitless injector operated at 45°C. Samples (2 g) were weighed and put in a 20 ml headspace glass, incubated at 40°C for 15 min with speed agitation of 500 rpm, and 500 μL of headspace gas was sampled with a heated syringe for analysis. Chromatographic separation was performed under isothermal conditions at 60°C for the sample. High purity nitrogen was used as the carrier gas with a varied flow rate (150 ml/min for 45°C column measurements).

Data analysis of GC-IMS was carried out using the functional software Laboratory Analytical Viewer and analysis software and three plug-ins Reporter, Gallery Plot, Dynamic PCA, and GC × IMS Library Search (G.A.S. Gesellschaft für analytische SensorsystemembH. Dortmund, Germany). The built-in NIST 2014 gas-phase retention index database and G.A.S IMS migration time database were used for two-dimensional qualitative analysis.

### GC-MS Determination Method

#### Sample Pretreatment of GC-MS Determination

The flavor of corn samples was analyzed with GC separation (7,890, Agilent, United States) and MS detection (5975C, Agilent, United States). According to Narisawa et al. (2017), certain modifications were made, and headspace (HS) solid-phase microextraction (SPME) followed by the GC-MS analysis was used for corn samples. DVB/CAR/PDMS coated SPME fibers (50/30μm, Supelco, United States) were selected (Godayol et al., 2011). Before utilizing, the SPME fiber was conditioned in the GC injection port according to Supelco instructions. Samples (6 g, crushed with a mortar) of different treatments were weighed (MP5002 Electronic balance: Shanghai Shunyu Hengping Scientific Instruments Co., Ltd., Shanghai, China) and placed in a 30 ml headspace glass. After equilibration for 20 min and headspace extraction at 80°C for 30 min, the SPME fiber was thermally desorbed at 250°C for 6 min in a splitless injection port of GC for analysis. After each run, the SPME fiber was cleaned by reheating for 20 min in the injection port.

#### GC-MS Determination

According to Narisawa et al. (2017), the concentrated gas was injected into the GC-MS system with a DB-5MS capillary column (60 mm × 0.32 mm × 1.0 μm) (Agilent, United States). Helium with 99.9995% purity was employed as the carrier gas at a flow rate of 0.8 ml/min. The injection temperature was 250°C. EI ionization was 70 eV and the ion source temperature was 230°C. The GC temperature was programmed from 40°C (holding for 2 min) to 180°C at 5°C/min, then to 250°C at a rate of 10°C/min, and held at 250°C for 10 min. The full-scan mode between 35 and 450 m/z was conducted for qualitative analysis, and the SIM model was used for quantitative analysis.

### Qualitative and Quantitative Analysis of Volatile Compounds

The retention indices (RI; positive and negative matching >800) of the components were determined using a series of n-alkanes (C_7_-C_30_) under identical GC conditions as described above, based on a comparison of mass spectra of sample compounds to the NIST spectral library (http://webbook.nist.gov/chemistry/). Volatile compounds were identified by comparing their retention times, linear retention indices, and mass spectra with those of the authentic standards analyzed under identical chromatographic conditions. Meanwhile, the relative content of each volatile compound was calculated by a ratio of the peak area of each component to the total area of peaks in typical GC-MS total ion chromatograms (TIC).

### ROAV Analysis of Volatile Compounds

The relative odor activity value (ROAV) is used to evaluate the contribution of individual compounds to the overall aroma (Wang et al., 2010). It was calculated as follows:

$$ROAV_{i}=\frac{C_{i}}{T_{i}}\times \frac{T_{\max}}{C_{\max}}\times 100$$

where C_i_ is the relative content of the volatile compound to be measured (%), T_i_ is the aroma threshold of the volatile compound to be measured in water (µg/kg), and T_max_ and C_max_ are the maximum of C_i_/T_i_ among all the compounds in the sample.

The relative odor activity value ranges from 0 to 100. Volatile compounds with ROAV ≥1 are considered as key odor compounds, of which >0.1 and smaller than 1 play an embellish role in aroma, of which <0.1 are potential aroma compounds.

### Sensory Evaluation

According to the characteristics of flavor compounds that have ROAV value greater than 10 in corns under nine kinds of different treatments, the corns were subjected to sensory evaluation by ten trained judges. The members of the panel were asked to indicate their preference for the volatile attributes of samples and overall acceptability based on a balanced 9-point hedonic rating. The scores were 8–9 “like very much,” 6–7 “like,” 5 “neutral,” 3–4 “dislike,” and 1–2 “dislike very much”. While the intensities of the various sensory attributes were evaluated based on a balanced 9-point hedonic rating, the 0–9 scores represent from “no perception or extremely weak” to “strong perception.” The sensory evaluation protocols complied with the guidelines of the declaration of Helsinki for studies on human subjects and was approved by the experiment ethics committee of Shanghai Institute of Technology. All subjects had studied the protocol and provided their written informed consent before the sensory evaluation.

### Data Processing and Statistical Analysis

Origin Pro 8.0 software (Origin Lab Inc., United States) was used for drawing. SPSS 16.0 (IBM, Armonk, NY, United States) was used to analyze the experimental data. All presented data are expressed as means ± SE. The statistical analyses were calculated using a one-way analysis of variance (ANOVA), and differences were considered significant at *p* < 0.05.

## Results and Discussion

### Flavor Differences in Corn Samples Under Different Treatments Detected by GC-IMS

**FIGURE 1:**
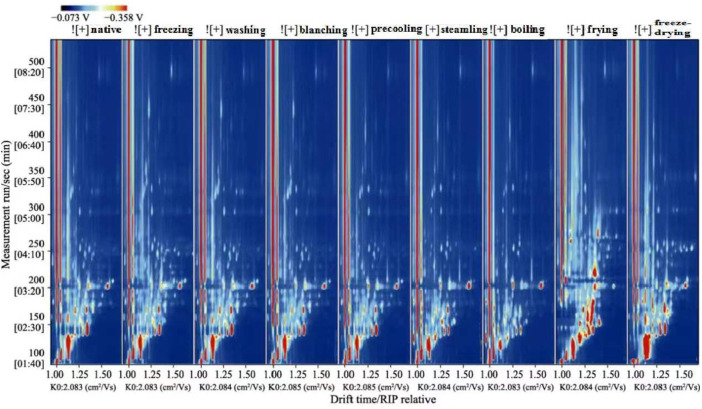
Two-dimensional spectrum of volatile compounds in corn under nine kinds of different treatments (top view).

In the topographic plots of GC-IMS spectra (Figure 1), the ordinate represented the retention time of the gas chromatography and the abscissa represented the ion migration time. The vertical line at the abscissa 1.0 was the RIP peak (reaction ion peak, normalized). Each point on both sides of the RIP peak represented a volatile organic compound, white means lower concentration, and red means higher concentration, that is, the darker the color, the higher the concentration. Combined with the aforementioned instructions and the two-dimensional top view of GC-IMS, the composition of volatile compounds among different samples could be compared intuitively.

Taking native corn as a reference, the difference analysis of all spectrums showed that there were obvious differences in flavor compounds. According to the spectrums and Supplementary Table S1, corns under nine kinds of treatments could be divided into four categories: native and washed corn; blanched, precooled, and frozen corn; steamed and boiled corn; fried and freeze-dried corn.

Compared with the native corn, the flavor spectrum of washed corn was similar to that of the native corn, and the concentration of flavor compounds was almost the same. It is speculated that there was not much difference between washed corn and native corn. Compared with native corn, the spectrums of blanched, precooled, and frozen corn had changed, but the effect on the flavor was relatively small. The flavor spectrums of blanched, precooled, and frozen corn were relatively similar. Blanching can promote the development of typical flavor components in corn. Studies have shown that alcohols were the main volatile components in native corn, and sulfur-containing compounds account for the largest proportion in blanched corn; these were the key volatile compounds that contribute to the aroma of blanched corn (Wiley, 1985). In addition, the content of soluble solids was one of the important indicators for evaluating food flavor, and it is an important factor in the edible quality of corn (Zampini et al., 2008). Blanched, precooled, and frozen corns were all processed for 2 min in boiling water in terms of treatment methods. Compared with blanched corn, precooled and frozen corn underwent short-time cold treatment. It was speculated that short-time heating and cold treatment had little effect on the solid content and flavor of corn. This speculation needs to be confirmed by subsequent experiments. In addition, compared with native corn, the flavor compounds of steamed and boiled corn decreased obviously, and the changes were more obvious. The difference in flavor between cooked corn and native corn may be due to both steaming and boiling belonging to wet heat treatment. Based on heat conduction and hydrodynamics, wet heat treatment could promote nonenzymatic oxidation and decomposition reactions of corn components. The adequate heat process could inactivate lipolytic enzymes and develop rich flavors. For example, wet heat treatment of oat flours reduced the levels of most flavors, particularly bitterness and astringency. The levels of hexanal and oat flavor increased, while most volatiles remained constant (Molteberg et al., 1996). It can be seen from the spectrum of fried and freeze-dried corns that the variety of volatile compounds was the most abundant, and the changes were more obvious compared with corn under other treatments. The volatile aroma compounds in fried corn might be caused by the concentration and threshold of volatile aroma compounds through the Maillard and caramelization reactions (Coleman et al., 1994). It was reported that the flavor formation in heat-treated food is often associated with the Maillard reaction, carbonyl compounds reacted with the amino compounds and provided special flavor under high-temperature conditions (Heydanek and Mcgorrin, 1981; Sacchetti et al., 2016). For example, Fors and Schlich (1989) found that the flavor composition of oats was mostly decided by the lipid concentration and processing, such as heating, milling, and roasting. High temperature affected both the Maillard reaction and lipid degradation, which probably formed more volatile compounds. During food processing, food microstructure played an important role in the retention and release of flavor compounds (Roos, 2003). The nature and quantity of flavor compound binding sites to the food matrix were influenced by microstructure, which determined the degree of flavor compound binding to the food matrix (Druaux and Voilley, 1997). Freeze-dried corn formed a porous honeycomb structure, and the integrity of the cell tissue structure and network pore morphology during the freeze-drying process had an important impact on the retention of volatile flavor compounds (Díaz-Maroto et al., 2003; Diaz-Maroto et al., 2004). Different treatments led to different volatile flavor compounds of corn, which is consistent with the research results of Sides et al. (2001) and Parker et al. (2000).

### Comparison of Flavor Fingerprints in Corn Samples Under Different Treatments by GC-IMS

**FIGURE 2:**
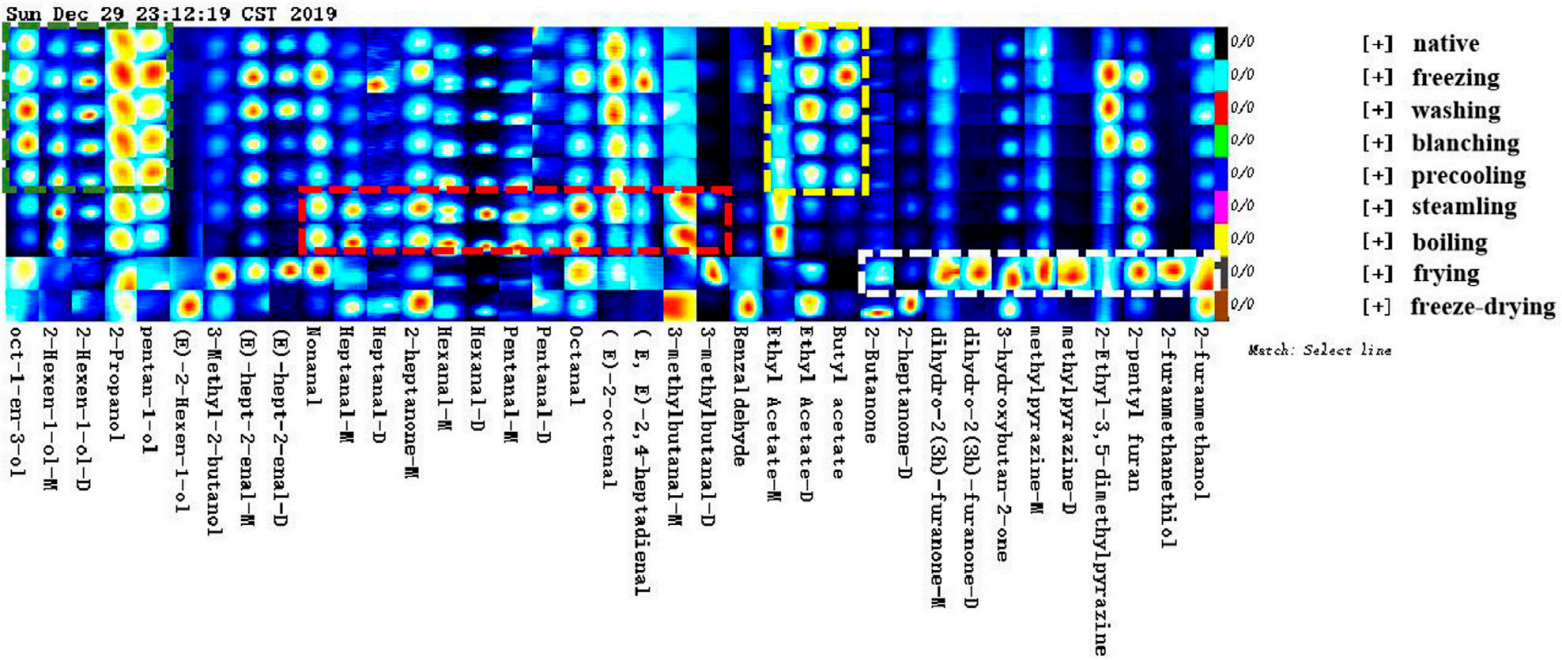
Gallery plot fingerprint of corn under nine kinds of different treatments.

In order to show the change law and relative concentration of volatile compounds in different samples more specifically and intuitively, the fingerprint spectrum (Figure 2) of volatile compounds was drawn using the Gallery Plot plug-in. Meanwhile, the differences in volatile compounds in different samples were more intuitively shown in Supplementary Table S1. Figure 2 showed the complete volatile organic compounds information of each sample and the difference in volatile organic compounds among the samples. The changes in flavor compounds in different samples were more obvious in the Gallery Plot fingerprint spectrum. Through longitudinal comparison, it can be seen intuitively that the concentration of different flavor compounds presented a certain law. The analysis of flavor fingerprints focused on the 37 volatile components that were qualitatively fast in this study. The same kinds of compounds were put together in order to analyze the changing laws of flavor compounds in samples under different treatments.

As shown in the green box, the concentration of the alcohols in native corn such as 1-octene-3-ol, trans-2-hexenol, pentanol, and 2-propanol reached the maximum, and remained unchanged significantly under the process of washing, blanching, and precooling, but the concentration of the main alcohols gradually decreased under the process of steaming, boiling, frying, and freeze-drying. This may be due to the continuous heating process that causes the small molecular alcohols with lower boiling points to continuously be taken away with the water vapor such as pentanol and 2-propanol. The research of Zeng et al. (2008) also confirmed that the concentration of small molecule alcohols with low boiling points decreased with the extension of cooking time. On the other hand, the formation of alcohol was related to the action of alcohol reductase. The production of alcohol was blocked due to the inactivation of the part of the alcohol reductase during heating, or alcohols might undergo esterification, condensation, cracking, and other reactions at higher temperatures.

As shown in the yellow box, the changing patterns of esters such as ethyl acetate and butyl acetate were similar to that of alcohols, which had the highest concentration in native corn, and gradually decreased under the processing of washing, blanching, precooling, and freezing, and the concentration reached the lowest after cooking. Similarly, the continuous high temperature made ethyl acetate being taken away with water vapor continuously. The boiling point of ethyl acetate was 77°C. Moreover, esters might undergo oxidation reactions during heating and decomposed to produce volatile carbonyl compounds such as ketones, aldehydes, and acids.

As shown in the red box, the concentration of various aldehydes such as octanal, 2-heptanal, nonanal, and 3-methylbutanal reached the maximum during the steaming and boiling process, indicating that a large number of aldehydes were generated. Corns contain linoleic acid, palmitic acid, and oleic acid. These fatty acids could be easily converted into aldehydes by oxidation reaction during the steaming and boiling process. Studies had shown that some aldehydes were also derived from the Maillard reaction of reducing sugars and amino acids, and the threshold of aldehydes was low, which made a relatively large contribution to the flavor of the sample (Yaylayan and Haffenden, 2003).

As shown in the white box, 2-heptanone, 2-butanone, 2-Ketones, 3-hydroxy-2-butanone, 2-methyl-pyrazine, and 2-ethyl-3,5-dimethyl-pyrazine with the highest concentration were produced during the frying process, Maillard reaction, lipid oxidation, and thermal degradation of sugar under high-temperature conditions were important ways to form the flavor of fried corn, especially pyrazine compounds with high concentration and low threshold value, which provided a strong nutty and roasting aroma for the overall flavor of fried corn (Coleman et al., 1994; Baker, 2002). It was more consistent with the research of Shi et al. (2018).

### Differences of Flavor Compounds in Corn Samples Under Different Treatments by GC-MS

Volatile compounds in corns were identified by GC-MS, the total concentration and types of volatile compounds differ depending on the treatments. A total of 152 flavor compounds were obtained by GC-MS analysis of native, washed, blanched, precooled, frozen, steamed, boiled, fried, and freeze-dried corn. Among them, the threshold of saturated alkanes was high and almost odorless, so it was not for a detailed analysis here. Among the flavor compounds analyzed by GC-MS (Supplementary Table S2), combined with the ROAV values in Supplementary Table S3, the following conclusions were drawn.

Among aldehydes, taking n-hexanal with high coincidence co-detected by two detection methods as an example, the ROAV value of n-hexanal in steamed, boiled, and fried corn was significantly higher than that in corn under other treatments. Under steaming treatment, heptanal with a little fruit aroma and trans-trans-2,4-nonadienal with lipid odor had higher ROAV values, and they were also detected in corn under other treatments. It was speculated that the reason might be the Maillard reaction at high temperature, so they were considered the characteristic flavor produced in steamed corn. It could be seen that the main flavor compounds were similar in steamed and boiled corn, but flavor compounds in steamed corn were more abundant than that in boiled corn. It was speculated that the water in corn would affect the flavor (Kramhöller et al., 1993; Parker et al., 2000; Acevedo et al., 2012). Among alcohols, taking 1-octene-3-ol as an example, the ROAV value in freeze-dried corn was the highest and decreased significantly under other treatments. It may be due to the oxidation of lipoxygenase on some fatty acids, reduction of alcohol dehydrogenase on some aldehydes (Fellman, 1997), and the hydrolysis of esters (Varlet et al., 2007). The concentration of heterocyclic compounds such as 2,5-dimethylpydrazine and 2-pentylfuran was the highest in fried corn. It may be due to the Maillard reaction and lipid hydrolysis reaction of fried corn that a large number of heterocyclic aroma compounds were formed, which could be regarded as the key aroma compounds of fried corn. All of them were consistent with the changing trend of alcohols, aldehydes, and heterocyclic compounds detected by GC-IMS alone. Esters were produced by the interaction of alcohols and free fatty acids produced by fat oxidation. Due to their low contribution to the aroma and low ROAV value, the difference in flavor was not obvious in corn under different treatments. Although the relative concentrations of diisobutyl phthalate, methyl 14-methyl pentadecanoate, and dibutyl phthalate were relatively low, they were almost found in corn under nine kinds of different treatments. In summary, the treatment methods for corn can be judged according to the total concentration of different compounds.

Combined with ROAV, the common characteristic flavor compounds in group 1 (native and washed corn) were screened as decylaldehyde and 1-octene-3-ol. The common characteristic flavor compounds in group 2 (blanched, precooled, and frozen corn) were screened as decylaldehyde and 1-octene-3-ol. The common characteristic flavor compounds in group 3 (steamed and boiled corn) were screened as n-hexanal, trans-2-nonanal, decylaldehyde, and 1-octene-3-ol, 2-pentyl furan. The characteristic flavor compounds of fried corn in group 4 were screened as n-hexanal, phenylacetaldehyde, trans-2-octenal, decylaldehyde, 2,5-dimethyl-pyrazine, and 2-pentylfuran. The characteristic flavor compounds of freeze-dried corn in group 4 were screened as nonanal, decylaldehyde, and 1-octene-3-ol. 2,5-dimethyl-pyrazine only existed in fried corn with a ROAV of 34.50. In addition, decylaldehyde constituted an abundant compound in all corn samples, obtained the ROAV of 11.70–100.00, and gave the highest contribution to the odor with fat and floral fragrances.

### Comparison of Flavor Compounds in Corn Samples Under Different Treatments by GC-IMS and GC-MS

Significant results were shown in Supplementary Tables S1, 2, but an accurate analysis could not be performed due to the large amount of data. Statistical analysis methods such as principal component analysis and cluster analysis may be more suitable for the analysis of this data. In addition, the Venn diagram combined with the ROAV method can explore the unique volatile compounds of corn under different treatments, which are applied to this study.

**FIGURE 3:**
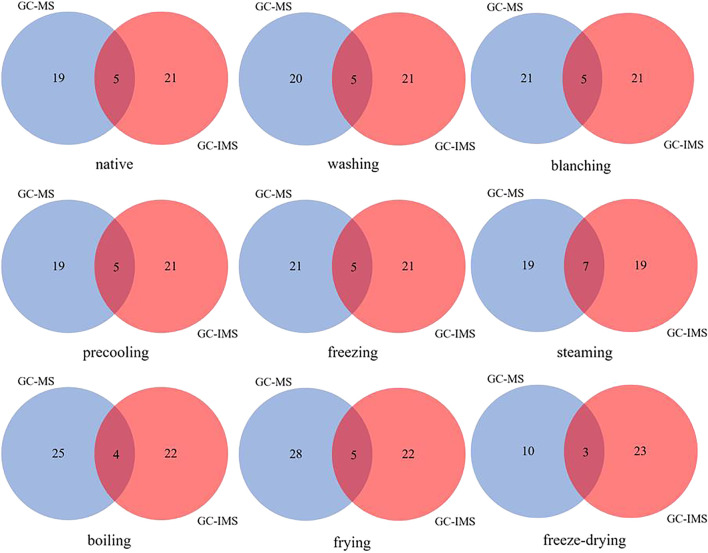
Wayne diagram of the flavor by GC-MS and GC-IMS under nine kinds of different treatments.

By comparing and analyzing the common flavor compounds of corn under different treatments, the Venn diagram (Figure 3) found that the common flavor compounds co-detected by GC-MS and GC-IMS were generally three to seven kinds, including n-hexanal, 1-octene-3-ol, octanol, 2-pentylfuran, and other compounds, which were speculated to be the key flavor compounds of corn. Specifically, nonanal was co-detected by GC-MS and GC-IMS under nine kinds of different treatments; n-hexanal, 2-pentylfuran, and 1-octene-3-alcohol were detected in corn under other treatments except for freeze-dried corn; benzaldehyde was co-detected by GC-MS and GC-IMS in freeze-dried, fried, and steamed corn; trans-2-octene was co-detected in corn under other treatments except for freeze-dried, fried, steamed, and boiled corn. The results are consistent with the conclusion that the concentration of aldehydes gradually decreased by GC-IMS alone, that is, the concentration of aldehydes in corn reached the lowest after steaming, boiling, frying, and freeze-drying. Therefore, it was feasible to detect the aroma of corn quickly by GC-IMS.

### Sensory Evaluation of Corn Samples Under Different Treatments

The flavor compounds with ROAV value greater than 1 in corn under nine kinds of different treatments were combined with their flavor characteristics (Supplementary Table S3). Six sensory descriptors were used to evaluate the aroma notes: sweet flowers, fat fragrance, mushroom hay, roasted potato or nuts, vegetable-like bean, and bitter almond, and the aroma profile was drawn based on sensory evaluation results.

**FIGURE 4:**
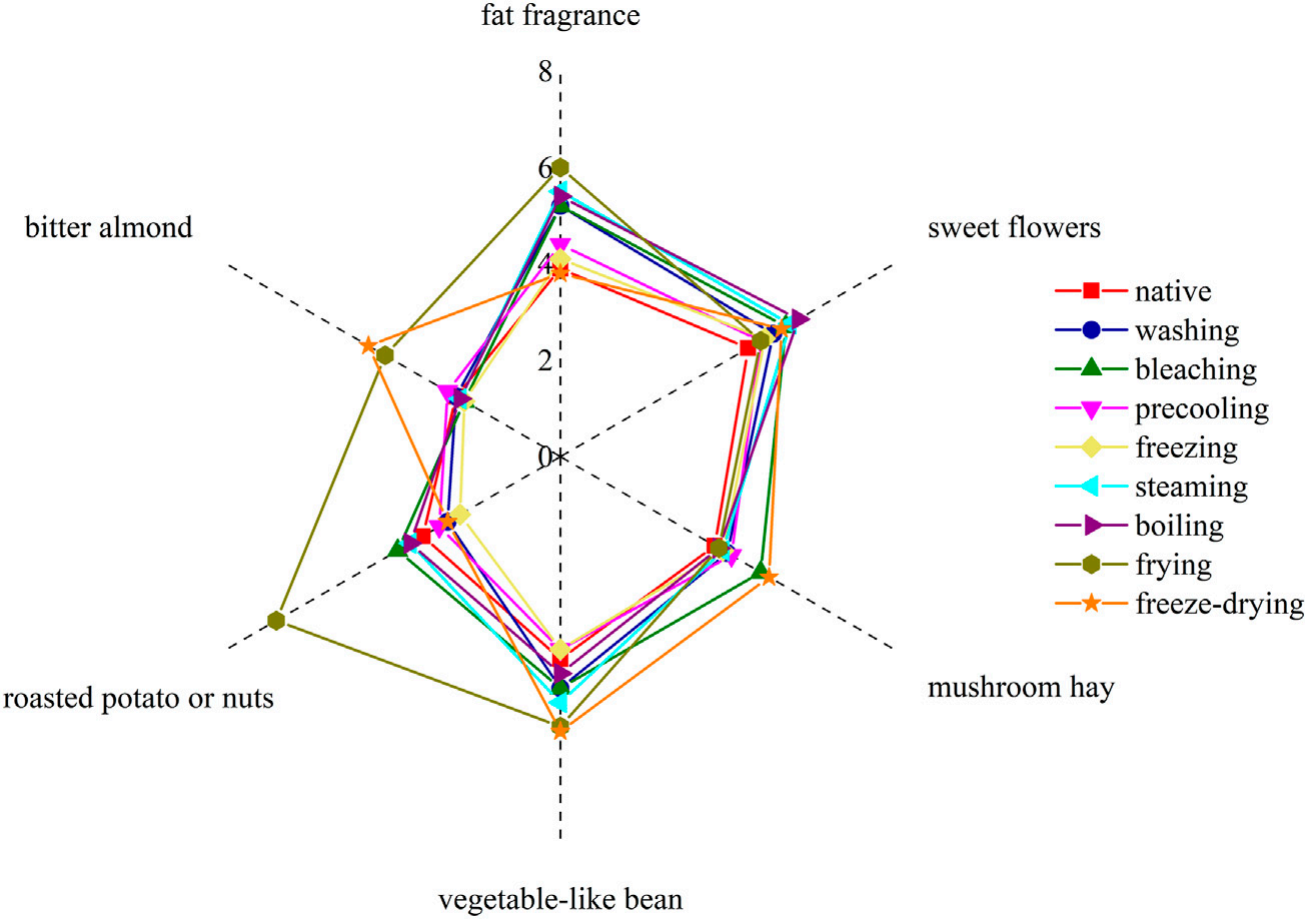
Aroma profile of corn under nine kinds of different treatments.

**FIGURE 5:**
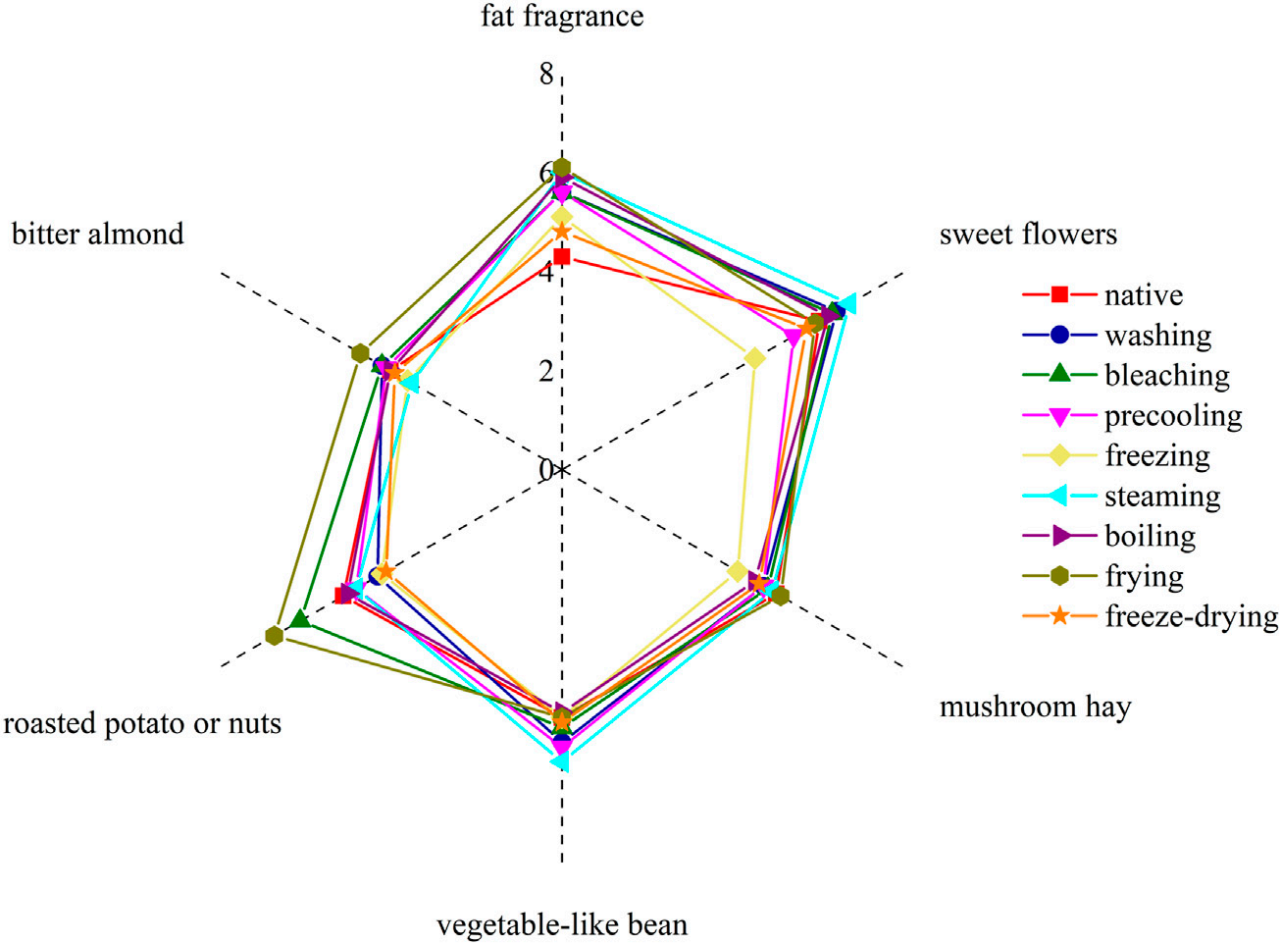
Sensory score of each note in corn under nine kinds of different treatments.

**FIGURE 6:**
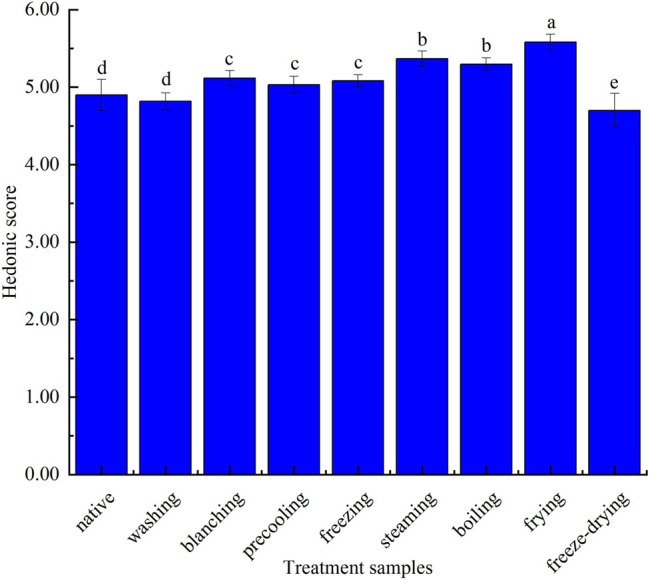
Hedonic score of corn under nine kinds of different treatments.

It can be seen from Figure 4 that the sweet flower note of the corn under nine kinds of treatments was similar. In detail, sweet flowers, vegetable-like beans, and fat fragrance notes were especially in steamed and boiled corn. The sweet flower note was mainly contributed by aldehydes such as nonanal, decanal, and phenylacetaldehyde, while the fat fragrance note was mainly caused by n-hexanal, trans-2-nonanal, and octanol. Vegetable-like bean note was mainly produced by 2-pentylfuran. Among them, the native corn aroma was mainly composed of sweet floral notes and vegetable-like bean notes because of the higher concentration of nonanal, decanal, and 2-pentylfuran. The bitter almond note caused by benzaldehyde in fried corn was more obvious, and pyrazines contributed greatly to roasted potato or nuts in fried corn. The freeze-dried corn had more mushroom hay notes due to the higher concentration of 1-octene-3-ol. The aroma profiles of boiled corn and steamed corn were the most similar, while those of washed corn, precooled corn, quick-frozen corn, and blanched corn were the most similar, which were basically consistent with the result of the fingerprint. Figure 5 showed the sensory score of each note in corn under nine kinds of treatments. Figure 6 showed the hedonic score of each corn sample. It could be seen from Figure 5 that the panel preferred fried corn, which was consistent with the result in Figure 6. It was no doubt that roasted potato or nuts in fried corn were most popular due to pyrazine aroma. The aroma profile and sensory score of steamed corn and boiled corn were similar, while the scores of the other five types of corn were slightly lower.

## Conclusion

Generally, the main flavor compounds in corn were compared under different treatments, but there were corresponding changes in the key flavor compounds of corn under different treatments. The key aroma compounds of ROAV ≥1 in corn under different treatments were n-hexanal, trans-2-octenal, nonanal, trans-2-nonanal, decanal, 1-octene-3-ol, 2-pentyl furan, etc. Among them, nonanal, 2-pentylfuran, and 1-octene-3-ol also proved to be the key flavor compounds of corn under different treatments by GC-MS analysis. It can be seen that the corn under different treatments mainly presented six characteristic aromas such as sweet flowers, fat fragrance, mushroom hay, roasted potato with nuts, vegetable-like bean, and bitter almond based on the sensory evaluation results, and the evaluators prefer the overall flavor of blanched corn, steamed corn, boiled corn, and fried corn. Meanwhile, the reason why corn was popular, and what kind of aroma panel liked in corn, what compounds presented the aroma all could be explained combined with GC-MS results. Therefore, this study established a rapid method to classify products by GC-IMS. On the other hand, according to the differences of key aroma compounds in corn under different treatments in this study, the next stage can be combined with the relevant processing technology to produce a specific flavor for further fine research in order to find the best way to process corn.

## Data Availability

The original contributions presented in the study are included in the article/Supplementary Material, further inquiries can be directed to the corresponding authors.
