# Supplementary material for: Analysis of Volatile Flavor Compounds of Corn Under Different Treatments by GC-MS and GC-IMS
Source: Front Chem. 2022 Jul 4;10:725208. doi: 10.3389/fchem.2022.725208 (PMC9290320; doi:10.3389/fchem.2022.725208)
Supplement: Supplementary file 1 [file Table1.DOCX]

**Tables**

Table 1 Peak volume of volatile compounds of corn under 9 kinds of different treatments via GC-IMS

| **#** | **Compounds** | Retention Time (s) | Retention Index | Drift Time (ms) | Peak volume of corn samples | | | | | | | | |
| --- | --- | --- | --- | --- | --- | --- | --- | --- | --- | --- | --- | --- | --- |
|  |  |  |  |  | native | washing | blanching | precooling | freezing | steaming | boiling | frying | freeze-drying |
| 1 | Nonanal. | 495.764 | 1105.70 | 1.4723 | 148.20±2.35h | 158.20±3.00g | 188.92±10.41e | 159.13±6.39f | 242.33±8.46b | 198.84±3.24d | 211.19±4.09c | 252.47±3.62a | 78.54±5.83i |
| 2 | 2-pentyl furan. | 336.63 | 990.10 | 1.25814 | 55.13±4.06i | 70.43±1.05g | 128.47±6.50e | 133.00±4.82d | 123.00±2.84f | 165.05±2.57a | 133.45±2.77c | 156.23±1.63b | 57.86±5.92h |
| 3 | 1-octene-3-ol. | 327.491 | 979.20 | 1.15925 | 122.13±1.27d | 157.04±3.23a | 154.20±3.26b | 109.89±9.31e | 138.63±4.60c | 82.55±1.04h | 61.23±1.03i | 106.93±5.84f | 86.40±3.g |
| 4 | Trans-2-heptenal. | 304.949 | 952.20 | 1.25666 | 165.72±1.01d | 213.99±2.11b | 162.80±11.53e | 175.10±10.01c | 219.94±5.82a | 159.39±2.73f | 98.39±3.85h | 137.06±3.81g | 95.41±3.95i |
| 5 | Heptyl aldehyde. | 262.301 | 901.00 | 1.32603 | 13.72±0.50h | 13.99±0.04g | 14.52±0.03e | 13.11±0.21i | 36.70±1.06a | 20.72±0.49d | 23.83±0.56c | 14.31±0.93f | 31.87±1.05b |
| 6 | 2-heptanone. | 253.162 | 888.70 | 1.26257 | 104.68±2.22e | 103.58±0.43f | 97.19±0.42h | 102.20±4.09g | 140.39±3.84d | 160.02±3.80b | 142.26±3.96c | 51.34±0.35i | 177.56±11.84a |
| 7 | Trans-2-hexenol. | 230.7 | 844.60 | 1.51908 | 91.51±1.01g | 118.99±3.00e | 141.56±3.42d | 145.04±4.79c | 108.08±3.90f | 179.59±4.86a | 175.74±4.42b | 75.38±1.74h | 53.54±3.95i |
| 8 | N-hexanal. | 203.347 | 791.00 | 1.56844 | 795.78±23.04g | 987.90±13.57c | 860.63±31.01e | 769.48±35.76h | 902.33±24.06d | 1381.69±26.95a | 1269.50±35.86b | 288.37±11.94i | 846.06±35.99f |
| 9 | Valeraldehyde. | 168.97 | 702.40 | 1.18439 | 39.89±0.89e | 37.77±0.57f | 26.80±0.08i | 28.09±1.21h | 44.77±4.00d | 48.61±1.05c | 52.09±1.84b | 29.79±0.31g | 64.25±5.83a |
| 10 | Ethyl acetate. | 142.013 | 593.20 | 1.09799 | 3316.59±45.66a | 2825.93±54.43b | 2059.39±72.74e | 1959.09±56.93f | 2234.81±38.52d | 949.35±43.80h | 564.78±17.94i | 1301.99±32.g | 2586.37±102.3c |
| 11 | 2-butanone. | 139.154 | 580.70 | 1.2492 | 162.74±3.46d | 173.06±2.57c | 146.86±11.67g | 142.23±6.02h | 157.99±3.95f | 141.20±2.93i | 159.62±3.95e | 291.03±3.95b | 324.19±22.85a |
| 12 | 2-propanol. | 122.817 | 509.10 | 1.08817 | 140.88±1.11h | 157.14±2.43g | 175.78±3.59e | 173.87±5.85f | 185.54±3.46d | 187.60±2.96c | 201.02±2.86b | 214.40±3.52a | 116.45±11.04i |
| 13 | Isovaleraldehyde. | 155.159 | 650.90 | 1.16037 | 33.43±0.09h | 52.71±1.42e | 37.07±2.51g | 39.29±3.21f | 29.30±4.73i | 103.97±1.93b | 123.47±4.95a | 59.64±1.04d | 72.68±3.74c |
| 14 | 3-hydroxy-2-butanone. | 172.968 | 713.00 | 1.3324 | 703.61±4.97f | 821.38±27.98c | 736.12±32.08d | 601.13±11.05h | 728.25±32.85e | 695.72±16.73g | 277.92±5.04i | 2501.31±58.93a | 1457.64±17.83b |
| 15 | 3-methyl-2-butanol. | 170.552 | 706.60 | 1.23642 | 105.98±2.92h | 131.55±2.75c | 123.02±1.86e | 114.94±4.07g | 128.35±4.90d | 120.20±5.62f | 72.12±1.24i | 347.27±3.97a | 174.24±2.95b |
| 16 | Benzaldehyde. | 307.87 | 955.70 | 1.14876 | 70.48±0.64f | 53.27±0.36g | 43.59±2.87i | 51.64±3.22h | 107.68±2.67b | 77.52±6.83d | 74.66±1.74e | 87.62±10.09c | 255.03±12.52a |
| 17 | Furfuryl alcohol. | 253.902 | 890.10 | 1.36407 | 81.18±0.33c | 78.69±1.04d | 64.29±0.43e | 53.68±5.87h | 62.47±4.53f | 56.16±4.87g | 34.74±0.64i | 125.67±2.95b | 148.58±2.95a |
| 18 | Trans-2-octenal. | 425.202 | 1055.60 | 1.33366 | 61.18±1.33c | 61.70±0.32d | 50.83±2.77f | 52.51±3.06e | 66.12±2.80a | 63.05±4.08b | 37.45±0.29h | 38.58±2.94g | 11.54±1.04i |
| 19 | trans,trans-2,4-Heptadienal. | 361.746 | 1010.60 | 1.19472 | 93.60±0.54g | 96.23±1.54f | 107.34±32.43c | 96.73±1.42e | 153.64±3.54a | 112.21±2.86b | 100.62±1.84d | 44.19±3.00h | 22.87±1.80i |
| 20 | Octanal. | 350.818 | 1002.80 | 1.40121 | 34.76±0.21i | 38.38±0.06h | 44.91±7.65e | 46.11±2.44d | 54.23±1.03c | 68.77±1.11a | 67.31±1.01b | 42.77±3.67f | 39.49±1.51g |
| 21 | γ-butyrolactone. | 276.04 | 917.50 | 1.08439 | 80.65±0.18c | 75.01±0.43d | 71.82±2.42e | 65.62±7.02g | 86.98±3.85b | 65.66±1.84f | 64.68±0.87h | 196.74±11.06a | 58.83±6.77i |
| 22 | 2-methylpyrazine. | 218.889 | 821.50 | 1.39796 | 143.23±2.93b | 129.85±1.11d | 121.03±11.97e | 113.51±11.65f | 132.74±11.63c | 102.36±1.64g | 87.35±1.95h | 258.66±20.03a | 57.44±3.94i |
| 23 | Butyl acetate. | 208.561 | 801.20 | 1.62241 | 128.98±3.44b | 128.36±2.59c | 91.30±8.55e | 101.30±10.96d | 176.36±9.95a | 34.37±0.54f | 24.53±1.68h | 2.04±0.01i | 33.09±2.77g |
| 24 | pentan-1-ol. | 189.394 | 756.40 | 1.25331 | 77.19±0.22g | 79.15±0.85e | 98.10±1.83c | 104.65±3.70a | 102.87±7.04b | 82.43±3.52d | 77.32±2.63f | 27.49±3.05h | 21.54±1.04i |
| 25 | Bran mercaptan. | 263.524 | 902.50 | 1.10384 | 14.70±0.04g | 17.34±0.01f | 16.04±0.64h | 11.39±0.09i | 28.51±1.83b | 23.60±1.95c | 20.15±0.95d | 338.16±21.85a | 19.37±3.60e |
| 26 | 2-Ethyl-3,5-dimethylpyrazin. | 441.528 | 1067.20 | 1.22518 | 124.75±3.21h | 299.84±17.42a | 278.73±34.04c | 145.87±11.06e | 297.68±21.53b | 137.53±0.98g | 114.60±11.84i | 197.34±3.07d | 139.37±11.09f |

All data represent the mean of triplicate determinations ±standard deviation. Means with different lowercase letter in the same column are significantly different (P <0.05).

Table 2 Volatile compounds identified of corn under 9 kinds of different treatments via GC-MS

| **#** | **Compounds ^a^** | Retention Time (min) ^b^ | Retention Index ^c^ | Reference Retention Index ^c^ | Relative content (%) | | | | | | | | |
| --- | --- | --- | --- | --- | --- | --- | --- | --- | --- | --- | --- | --- | --- |
|  |  |  |  |  | native | washing | blanching | precooling | freezing | steaming | boiling | frying | freeze-drying |
| 1 | N-hexanal. | 5.313 | 1070 | 1072 | 6.92±3.04e | 5.60±2.11f | 9.05±3.44d | 14.64±5.63c | 5.49±1.00g | 11.53±4.01b | 12.50±3.78a | 3.39±1.00h | 0.00±0.00i |
| 2 | Heptaldehyde. | 8.071 | 953 | 956 | 0.00±0.00b | 0.00±0.00b | 0.00±0.00b | 0.00±0.00b | 0.00±0.00b | 0.99±0.47a | 0.00±0.00b | 0.00±0.00b | 0.00±0.00b |
| 3 | Benzaldehyde. | 9.833 | 1509 | 1510 | 0.00±0.00d | 0.00±0.00d | 0.00±0.00d | 0.00±0.00d | 0.00±0.00d | 4.57±1.01c | 0.00±0.00d | 5.72±1.08b | 19.32±3.67a |
| 4 | Phenylacetaldehyde. | 12.414 | 1620 | 1618 | 0.00±0.00g | 1.18±0.49e | 0.00±0.00g | 1.79±1.01c | 1.18±0.40f | 1.59±0.84b | 1.36±0.48d | 2.05±0.95a | 0.00±0.00g |
| 5 | Trans-2-octenal. | 12.86 | 1010 | 1013 | 2.82±0.71c | 2.06±0.05e | 2.88±1.09d | 3.93±1.44b | 1.57±0.89f | 2.98±1.00a | 0.00±0.00h | 0.78±0.10g | 0.00±0.00h |
| 6 | Nonanal. | 14.273 | 1386 | 1390 | 14.10±5.06g | 18.58±7.08f | 23.87±11.28e | 29.64±13.07d | 14.51±3.05h | 28.23±10.09b | 32.07±8.01a | 8.69±3.04i | 29.39±6.21c |
| 7 | Trans-2-nonanal. | 15.915 | 1051 | 1052 | 2.56±1.07b | 0.00±0.00f | 2.06±0.05d | 2.86±0.67c | 0.00±0.00f | 3.38±1.03a | 1.90±0.64e | 0.00±0.00f | 0.00±0.00f |
| 8 | Trans-2-decenal. | 18.828 | 1650 | 1655 | 0.00±0.00c | 0.00±0.00c | 0.00±0.00c | 0.00±0.00c | 0.00±0.00c | 0.80±0.11a | 0.54±0.05b | 0.00±0.00c | 0.00±0.00c |
| 9 | trans,trans-2,4-Decadien-1-al. | 20.333 | 1218 | 1220 | 0.00±0.00b | 0.00±0.00b | 0.00±0.00b | 0.00±0.00b | 0.00±0.00b | 7.75±2.56a | 0.00±0.00b | 0.00±0.00b | 0.00±0.00b |
| 10 | Decyl aldehyde. | 17.249 | 1500 | 1498 | 3.59±0.42g | 17.11±3.96a | 9.05±2.22b | 4.29±1.00h | 8.24±2.00e | 7.55±4.21c | 8.15±2.07d | 2.40±1.09i | 5.15±2.02f |
| 11 | trans,trans-2,4-Nonadienal. | 17.495 | 1712 | 1715 | 0.00±0.00b | 0.00±0.00b | 0.00±0.00b | 0.00±0.00b | 0.00±0.00b | 0.60±0.01a | 0.00±0.00b | 0.00±0.00b | 0.00±0.00b |
| 12 | Nerol. | 17.895 | 1230 | 1231 | 0.00±0.00b | 0.00±0.00b | 0.00±0.00b | 0.00±0.00b | 0.00±0.00b | 3.38±1.76a | 0.00±0.00b | 0.00±0.00b | 0.00±0.00b |
| 13 | Octanol. | 13.278 | 1552 | 1554 | 12.82±2.66a | 10.03±3.01d | 10.29±2.07c | 12.50±3.04b | 8.24±2.54e | 0.00±0.00h | 5.98±1.34f | 1.77±0.88g | 0.00±0.00h |
| 14 | 1-octene-3-ol. | 10.434 | 1453 | 1456 | 11.03±3.06a | 4.72±0.29f | 5.76±2.36d | 7.86±1.12c | 5.49±2.11e | 1.79±0.30h | 2.99±0.68g | 0.00±0.00i | 8.55±1.44b |
| 15 | 2,6-Octadien-1-ol, 3,7-dimethyl-, (Z)- | 17.895 | 1844 | 1847 | 0.00±0.00b | 0.00±0.00b | 0.00±0.00b | 0.00±0.00b | 0.00±0.00b | 3.38±1.00a | 0.00±0.00b | 0.00±0.00b | 0.00±0.00b |
| 16 | Cedrol. | 27.588 | 2068 | 2069 | 0.00±0.00e | 0.00±0.00e | 0.41±0.06b | 1.43±0.06a | 0.00±0.00e | 0.40±0.00c | 0.27±0.20d | 0.00±0.00e | 0.00±0.00e |
| 17 | 3-octene-2-one. | 12.288 | 1344 | 1345 | 2.05±0.07a | 0.00±0.00c | 0.00±0.00c | 0.00±0.00c | 1.57±0.11b | 0.00±0.00c | 0.00±0.00c | 0.00±0.00c | 0.00±0.00c |
| 18 | 2-nonanone. | 13.907 | 1386 | 1390 | 3.59±0.45a | 0.00±0.00g | 2.88±0.45c | 3.21±1.00b | 2.35±1.10d | 0.99±0.11f | 1.09±0.06e | 0.00±0.00g | 0.00±0.00g |
| 19 | Methyl octyl ketone | 16.888 | 1193 | 1193 | 0.26±0.10d | 0.00±0.00e | 0.41±0.11a | 0.36±0.00b | 0.00±0.00e | 0.00±0.00e | 0.27±0.04c | 0.00±0.00e | 0.00±0.00e |
| 20 | Alpha- ionone. | 23.291 | 1415 | 1414 | 0.00±0.00d | 0.29±0.06b | 0.00±0.00d | 0.36±0.01a | 0.00±0.00 | 0.20±0.01c | 0.00±0.00d | 0.00±0.00d | 0.00±0.00d |
| 21 | BETA- dihydroionone. | 23.56 | 1430 | 1428 | 0.26±0.05e | 0.29±0.06c | 0.41±0.01a | 0.36±0.01b | 0.00±0.00g | 0.20±0.00f | 0.27±0.11d | 0.00±0.00g | 0.00±0.00g |
| 22 | 2,3-Dihydro-3,5-dihydroxy-6-methyl-  4(H)-pyran-4-one. | 15.486 | 1130 | 1131 | 0.00±0.00b | 0.00±0.00b | 0.00±0.00b | 0.00±0.00b | 0.00±0.00b | 0.00±0.00b | 0.00±0.00b | 7.84±3.48a | 0.00±0.00b |
| 23 | 2-Cyclohexen-1-one, 2-methyl-5-(1-methylethenyl)-, (S)- | 18.376 | 1241 | 1243 | 0.00±0.00b | 0.00±0.00b | 0.00±0.00b | 0.00±0.00b | 0.00±0.00b | 0.80±0.13a | 0.00±0.00b | 0.00±0.00b | 0.00±0.00b |
| 24 | Alpha-iso-methylionone. | 23.286 | 1470 | 1472 | 0.26±0.05d | 0.00±0.00f | 0.41±0.02a | 0.00±0.00f | 0.39±0.06b | 0.00±0.00f | 0.27±0.00c | 0.07±0.00e | 0.00±0.00f |
| 25 | 6, 10-dimethyl-5,9-undecadiene-2-one. | 23.811 | 1855 | 1853 | 2.05±0.21a | 0.59±0.04f | 1.23±0.45b | 0.00±0.00h | 0.78±0.06e | 0.99±0.06c | 0.82±0.43d | 0.42±0.05g | 0.00±0.00h |
| 26 | N-heptadecane. | 29.603 | 1674 | 1677 | 0.00±0.00e | 0.29±0.02c | 0.82±0.23a | 0.00±0.00e | 0.39±0.00b | 0.00±0.00e | 0.82±0.05a | 0.07±0.01d | 0.00±0.00e |
| 27 | Ethyl Laurate. | 27.257 | 1825 | 1828 | 0.00±0.00d | 0.29±0.01a | 0.00±0.00d | 0.00±0.00d | 0.00±0.00d | 0.00±0.00d | 0.27±0.10b | 0.21±0.07c | 0.00±0.00d |
| 28 | Diisobutyl phthalate. | 32.715 | 2420 | 2421 | 1.79±0.14b | 0.29±0.01e | 0.00±0.00g | 4.64±1.00a | 1.18±0.43d | 0.00±0.00g | 1.36±0.21c | 0.21±0.04f | 0.00±0.00g |
| 29 | Methyl 14-methyl pentadecanoate. | 33.488 | 1928 | 1926 | 0.00±0.00d | 0.00±0.00d | 0.82±0.08a | 0.00±0.00d | 0.00±0.00d | 0.00±0.00d | 0.27±0.10c | 0.28±0.10b | 0.00±0.00d |
| 30 | Dibutyl phthalate. | 34.014 | 990 | 988 | 0.00±0.00h | 0.88±0.12e | 3.70±0.94b | 5.71±0.60a | 0.00±0.00h | 2.39±0.78c | 2.17±1.00d | 0.64±0.13f | 0.23±0.05g |
| 31 | Ethyl palmitate. | 34.358 | 2001 | 1997 | 1.54±0.07f | 1.18±0.11d | 2.88±1.01b | 3.57±1.12a | 1.18±0.20d | 1.79±0.50e | 2.45±0.43c | 0.28±0.05g | 0.12±0.03h |
| 32 | Isopropyl Myristate. | 32.017 | 2240 | 2243 | 0.26±0.03c | 0.29±0.02b | 0.82±0.05a | 0.00±0.00d | 0.00±0.00d | 0.00±0.00d | 0.00±0.00d | 0.00±0.00d | 0.00±0.00d |
| 33 | Tetradecane. | 22.507 | 1411 | 1409 | 0.51±0.11f | 0.88±0.02b | 1.23±0.27a | 0.71±0.21e | 0.78±0.23d | 0.00±0.00h | 0.82±0.05c | 0.35±0.10g | 0.00±0.00h |
| 34 | Benzene, 1-methoxy-4-(1-propenyl)- | 19.532 | 1284 | 1285 | 0.00±0.00b | 0.00±0.00b | 0.00±0.00b | 0.00±0.00b | 0.39±0.00a | 0.00±0.00b | 0.00±0.00b | 0.00±0.00b | 0.00±0.00b |
| 35 | Butylated Hydroxytoluene. | 25.391 | 2243 | 2245 | 3.08±1.56a | 2.95±0.32b | 0.41±0.03f | 0.71±0.01d | 0.78±0.15c | 0.60±0.00e | 0.00±0.00h | 0.07±0.00g | 0.00±0.00h |
| 36 | N-hexadecane. | 27.365 | 1560 | 1561 | 1.28±0.69b | 0.59±0.06e | 1.23±0.20c | 0.00±0.00g | 1.18±0.43d | 0.00±0.00g | 1.36±0.89a | 0.14±0.00f | 0.00±0.00g |
| 37 | 4-vinyl-2-methoxyphenol. | 20.287 | 2162 | 2165 | 2.31±0.45d | 1.18±0.67f | 2.06±0.68e | 0.00±0.00g | 2.35±1.01c | 4.37±1.08a | 3.26±1.00b | 0.00±0.00g | 0.00±0.00g |
| 38 | (+)-limonene. | 11.944 | 1201 | 1205 | 0.00±0.00b | 0.00±0.00b | 0.00±0.00b | 0.00±0.00b | 0.00±0.00b | 0.00±0.00b | 0.00±0.00b | 0.00±0.00b | 26.46±3.66a |
| 39 | 2,5-dimethylpyrazine. | 8.305 | 910 | 913 | 0.00±0.00b | 0.00±0.00b | 0.00±0.00b | 0.00±0.00b | 0.00±0.00b | 0.00±0.00b | 0.00±0.00b | 14.91±3.07a | 0.00±0.00b |
| 40 | 2-ethyl-6-methylpyrazine. | 10.983 | 990 | 994 | 0.00±0.00b | 0.00±0.00b | 0.00±0.00b | 0.00±0.00b | 0.00±0.00b | 0.00±0.00b | 0.00±0.00b | 2.54±0.67a | 0.00±0.00b |
| 41 | 2-ethenyl-6-methyl pyrazine. | 11.538 | 1021 | 1022 | 0.00±0.00b | 0.00±0.00b | 0.00±0.00b | 0.00±0.00b | 0.00±0.00b | 0.00±0.00b | 0.00±0.00b | 5.09±2.05a | 0.00±0.00b |
| 42 | 3-ethyl-2,5-methylpyrazine. | 13.507 | 1077 | 1080 | 0.00±0.00b | 0.00±0.00b | 0.00±0.00b | 0.00±0.00b | 0.00±0.00b | 0.00±0.00b | 0.00±0.00b | 29.75±5.83a | 7.73±3.01b |
| 43 | 2-acetyl pyrrole. | 12.922 | 1955 | 1952 | 0.00±0.00b | 0.00±0.00b | 0.00±0.00b | 0.00±0.00b | 0.00±0.00b | 0.00±0.00b | 0.00±0.00b | 4.52±1.09a | 0.00±0.00b |
| 44 | 2-pentyl furan. | 10.823 | 1232 | 1235 | 16.41±3.05a | 9.44±1.67d | 14.81±1.23c | 0.00±0.00h | 15.29±3.43b | 7.36±3.07g | 8.97±4.07e | 7.77±2.04f | 0.00±0.00h |
| 45 | M-xylene. | 7.189 | 870 | 867 | 0.00±0.00d | 0.00±0.00d | 1.23±0.20b | 0.00±0.00d | 1.18±1.02c | 0.00±0.00d | 0.00±0.00d | 0.00±0.00d | 3.04±1.03a |
| 46 | Methoxyphenyl oxime. | 8.174 | 960 | 962 | 8.72±2.61c | 21.24±3.09b | 0.00±0.00e | 0.00±0.00e | 25.49±4.05a | 0.00±0.00e | 8.70±4.33d | 0.00±0.00e | 0.00±0.00e |
| 47 | 2-Acetylthiazole | 11.658 | 1016 | 1015 | 1.79±0.44a | 0.00±0.00f | 1.23±0.32d | 1.43±0.11b | 0.00±0.00f | 1.39±0.33c | 1.09±0.60e | 0.00±0.00f | 0.00±0.00f |

All data represent the mean of triplicate determinations ±standard deviation. Means with different lowercase letter in the same column are significantly different (P <0.05).

a Volatile compounds identified in HS-SPME-GC-MS.

b Retention time calculated on DB-5MS column.

c Retention index calculated on DB-5MS column. Compound: compounds with positive and negative matching >800. RI and Relative content: the mean values of parallel experiment.

Table 3 Main odor-active compounds (ROAV ≥ 1) of corn under 9 kinds of different treatments via GC-MS

| Compounds ^a^ | Aroma Threshold  (µg/kg) | ROAV | | | | | | | | |
| --- | --- | --- | --- | --- | --- | --- | --- | --- | --- | --- |
|  |  | native | washing | blanching | precooling | freezing | steaming | boiling | frying | freeze-drying |
| N-hexanal. | 4.5 ^b^ | 0.51 | 0.73 | 2.22 | 1.59 | 0.53 | 3.39 | 3.40 | 3.13 | 0.00 |
| Heptyl aldehyde. | 3 ^b^ | 0.00 | 0.00 | 0.00 | 0.00 | 0.00 | 0.44 | 0.00 | 0.00 | 0.00 |
| Benzaldehyde. | 320 ^b^ | 0.00 | 0.00 | 0.00 | 0.00 | 0.00 | 0.02 | 0.00 | 0.07 | 0.11 |
| Phenylacetaldehyde. | 4 ^c^ | 0.00 | 0.17 | 0.00 | 1.04 | 0.13 | 0.53 | 0.42 | 2.13 | 0.00 |
| Trans-2-octenal. | 3 ^d^ | 0.30 | 0.40 | 1.06 | 3.05 | 0.22 | 1.31 | 0.00 | 1.08 | 0.00 |
| Nonanal. | 40 ^b^ | 0.14 | 0.27 | 0.66 | 1.73 | 0.19 | 0.93 | 0.98 | 0.90 | 1.43 |
| Trans-2-nonanal. | 0.08 ^d^ | 10.48 | 0.00 | 28.41 | 83.33 | 0.00 | 55.92 | 29.17 | 0.00 | 0.00 |
| trans,trans-2,4-Decadien-1-al. | 0.2 ^e^ | 0.00 | 0.00 | 0.00 | 0.00 | 0.00 | 51.32 | 0.00 | 0.00 | 0.00 |
| Decyl aldehyde. | 0.1^e^ | 11.73 | 100.00 | 100.00 | 100.00 | 35.18 | 100.00 | 100.00 | 100.00 | 100.00 |
| trans,trans-2,4-Nonadienal. | 0.05 ^d^ | 0.00 | 0.00 | 0.00 | 0.00 | 0.00 | 15.79 | 0.00 | 0.00 | 0.00 |
| Octanol. | 100 ^d^ | 0.06 | 0.06 | 0.11 | 0.29 | 0.05 | 0.00 | 0.07 | 0.07 | 0.00 |
| 1-octene-3-ol. | 1 ^f^ | 3.60 | 2.76 | 6.36 | 8.33 | 2.35 | 2.37 | 3.67 | 0.00 | 16.59 |
| Cedrol. | 0.5 ^d^ | 0.00 | 0.00 | 0.91 | 6.67 | 0.00 | 1.05 | 0.67 | 0.00 | 0.00 |
| 3-octene-2-one. | 0.0067 ^e^ | 100.00 | 0.00 | 0.00 | 0.00 | 100.00 | 0.00 | 0.00 | 0.00 | 0.00 |
| 2,5-Dimethyl pyrazine. | 1.8 ^e^ | 0.00 | 0.00 | 0.00 | 0.00 | 0.00 | 0.00 | 0.00 | 34.50 | 0.00 |
| 2-pentyl furan. | 6 ^f^ | 0.88 | 0.92 | 2.73 | 0.00 | 1.07 | 1.63 | 1.84 | 5.40 | 0.00 |

a Volatile compounds identified in HS-SPME-GC-MS.

b The threshold of volatile compounds in water referred in the literature (Zhu, et al., 2016).

c The threshold of volatile compounds in water referred in the literature (Zhu, et al., 2019).

d The threshold of volatile compounds in water referred in the literature (Wang, et al., 2020).

e The threshold of volatile compounds in water referred in the literature (Gemert, 2003).

f The threshold of volatile compounds in water referred in the literature (Liu, et al., 2019).
